# “Symptomatic” melanoma brain metastases: A call for clear definitions and adoption of standardized tools

**DOI:** 10.1016/j.ejca.2024.114202

**Published:** 2024-07-06

**Authors:** Emilie Le Rhun, Michael Weller, Carey Anders, James Larkin, Jing Li, Nelson S Moss, Hussein Tawbi, Reinhard Dummer

**Affiliations:** a Department of Neurosurgery, Clinical Neuroscience Center, University Hospital and University of Zurich, Zurich, Switzerland; b Department of Neurology, Clinical Neuroscience Center, University Hospital and University of Zurich, Zurich, Switzerland; c Duke Cancer Institute, Durham, United Kingdom; d The Royal Marsden NHS Foundation Trust, London, United Kingdom; e Department of Radiation Oncology, University of Texas MD Anderson Cancer Center, Houston, TX, USA; f Department of Neurosurgery and Brain Metastasis Center, Memorial Sloan Kettering Cancer Center, New York, NY, USA; g Department of Melanoma Medical Oncology, Division of Cancer Medicine, The University of Texas MD Anderson Cancer Center, Houston, TX, USA; h Department of Dermatology, University Hospital and University of Zurich, Zurich, Switzerland

**Keywords:** Assessment, Cancer, Clinical, Evaluation, Neurological, Response

## Abstract

With improved systemic treatment and prolonged survival even with metastatic disease, diagnosing, treating, and monitoring brain metastases has become a central topic in the care of patients with melanoma. Patients with brain metastases from melanoma are typically excluded from pivotal clinical trials. When allowed, inclusion and exclusion criteria are rather selective and do not reflect the larger population of melanoma patients with brain metastases who frequently present with neurological symptoms and signs and require steroid medications. Moreover, the lack of consensus on reporting symptomatic brain involvement complicates the interpretation and implications of trial results for the overall population of patients with melanoma and brain metastasis. Here, we review the evidence regarding brain metastasis from melanoma and discuss the challenges of longitudinal neurological clinical assessments, including tools to capture cognition and quality of life. Finally, we propose the adoption of standardized tools to interpret neurological deficits in patients with melanoma and brain metastases and to assess the neurological status in the context of clinical trials.

## 1. Introduction

The incidence of CNS metastases remains high in melanoma, with a lifetime risk of 20–50 %, and 90 % at autopsy [1–4]. Although immune checkpoint inhibitors and targeted therapies demonstrate activity, median survival in patients with melanoma brain metastases remains below 15 months [5,6]. They are generally excluded from clinical trials, and when included, selective inclusion criteria are applied [7]. While several prospective trials have shown activity for immune and targeted therapies, the interpretation of such studies for patients with brain metastases in daily practice remains uncertain.

Melanoma brain metastases are therefore an excellent paradigm to illustrate opportunities and challenges of a multidisciplinary approach [1,8,9]. Many questions complicate the clinical management of these patients: (i) Challenges include sensitivity for and interpretation of small lesions on standard-thickness MRI, notably in the elderly. Detection of such lesions causes insecurity for physicians, patients and caregivers, a situation increasingly encountered with the implementation of screening for brain metastases. (ii) Optimized sequencing of systemic and local treatments and their combination remain unresolved. (iii) Response monitoring of treated brain lesions is challenging, notably with treatments like immune checkpoint inhibitors and radiosurgery that may cause treatment-related changes difficult to distinguish from tumor progression [10]. (iv) Lack of standardized validated tools for reporting neurological symptoms and signs limits understanding clinical trial results. The use of validated tools is crucial as some patients with brain metastases present with clinical deterioration not otherwise accounted for by imaging findings. (v) A consensus definition of asymptomatic versus symptomatic patients is lacking [11] and different criteria have been employed to define the brain metastasis patient population without versus with neurological deficit [7,12,13].

Here we review the data from prospective clinical trials in “symptomatic” patients with melanoma brain metastasis and focus on challenges assessing neurological function, cognition, quality of life and imaging and propose the adoption of validated tools to overcome current shortcomings.

## 2. Defining symptomatic versus asymptomatic patients in clinical trials

Approximately 30 % of melanoma patients with brain metastases present with neurological deficits at diagnosis [6]. Their proportion will become smaller with the broader implementation of neuroimaging for screening. Some trials have enrolled only patients with asymptomatic brain metastases (Note S1, Table 1). Different definitions have been used to define symptomatic brain metastases from melanoma in clinical trials. The goal has been to identify populations at risk of early clinical deterioration, including likelihood to require steroids. Symptomatic patients were defined as with (i) symptoms related to brain metastases [12], or (ii) with stable neurologic symptoms and signs related to brain metastases or with systemic steroid intake [14] or (iii) receiving more than 2 mg/day of dexamethasone equivalent, requiring anti-epileptic drugs, or presenting with brain-related symptoms and signs [13].

The presence of neurological symptoms and signs per se could explain overall worse outcomes. Further, corticosteroids antagonize the efficacy of immune checkpoint inhibitors. In the Checkmate 204 trial, the overall intracranial response rate was 44 % in asymptomatic patients versus 17 % in symptomatic patients [7]. A recent analysis of COMBI-MB analyzed data from all four cohorts based on corticosteroid use at baseline at baseline among other prognostic factors. Indeed, patients treated by steroids at baseline seemed to have a much more limited benefit from targeted therapy (Table 2) [15]. Therefore, the presence of large enough metastases to cause symptoms, including inflammation, may induce infiltration of suppressive immune cell populations that, in addition to requiring steroid therapy, can undermine the clinical activity of systemic agents [16]. Future therapeutic strategies may also be based on a more thorough characterization and understanding of the microenvironment in a site-specific manner which may allow targeted approaches not only for the tumor, but also its microenvironment.

Furthermore, and adding additional complexity, clinical oncology trials have often tried to distinguish *active from inactive* brain metastases which does not appear to be a resilient concept. Mostly, inactive brain metastases are defined as metastases that have been previously treated and are not currently enlarging. These lesions may correspond to scars from previous treatment, and monitoring their evolution appears to measure more the prophylactic activity of an intervention, rather than treatment, and such questions should be tested with appropriate study designs. Importantly, such lesions although referred to as inactive are captured only at a single landmark, and their further natural evolution is difficult to predict. Given the challenges of identifying progression and the lack of standardization of time intervals from previous treatments, the concept of *active* versus *inactive* brain metastases should probably be abandoned.

## 3. Clinical assessment

There are specific challenges associated with the clinical assessment of patients with melanoma and brain metastases. Primary oncology physicians are almost never neurologists, but organ specialists with a medical oncology or dermatology background. However, the clinical features of brain metastases at diagnosis and during the follow-up may require experience in neurological evaluation. Neurologic impairment can fall into the categories of focal deficits, e.g., paresis related to tumor destruction or edema in the corticospinal tract, global symptoms, e.g., related to intracranial hypertension owing to obstructive hydrocephalus, or functional deficits, e.g., related to encephalopathy caused by medications or seizures. Importantly, in patients with more than one lesion, assignment of symptoms and signs referable to a specific tumor is often necessary and requires both neuroradiologic interpretation and an understanding of structure-function relationships in the nervous system. Clinical acumen accounting for potential extracranial causes of impairment e.g. from spinal metastasis, peripheral neuropathy, or direct joint involvement is also necessary.

At the diagnosis of CNS metastases, a critical issue is the recognition and interpretation of pre-existing neurological symptoms or signs that may be related to toxicity from prior oncological treatments, such as cognitive deficits induced by radiotherapy or systemic treatment (“chemobrain”), neuropathy induced by chemotherapy or immunotherapy, or myopathy induced by steroid use. Neurological symptoms or signs existing prior to the diagnosis of brain metastases may also be related to other concomitant neurological diseases. Of note, rarely, un-related neurological diseases may be diagnosed during a patient’s course with clinically identified brain metastases. In case of doubt, if symptoms and signs are not readily explained by the brain metastases, a neurological assessment must be obtained to evaluate potential differential diagnoses.

Transient neurological deficits may also be observed, e.g., seizures or post-ictal (“Todd’s”) paralysis, or confusion. In the context of brain tumors, patients may need some recovery time from an acute convulsive or nonconvulsive seizure, and the neurological assessment should probably be repeated and retrospectively corrected once the patient has recovered.

The consumption and the doses of co-medications at the time of each neurological evaluation that may influence the neurological status should be collected. These medications include not only steroids which may mask symptoms, but also analgesics, which may reduce headache or induce nausea, or anti-epileptic drugs.

Corticosteroids including dexamethasone are frequently used in patients with brain metastasis because of their reliable impact on symptom control. However, the use of corticosteroids is associated with reduced efficacy of immunotherapy and several other important adverse effects. There is an obvious need for alternative edema-controlling medications that do not suppress immune reaction in the brain. A promising candidate in this context is bevacizumab [17]. The inherent risk of bleeding of melanoma brain metastases requires prospective monitoring for this potential adverse event. This would also include concurrent interventions which increase the risk of hemorrhagic complications, e.g., anticoagulation, as venous thromboembolic disease is common among patient with melanoma. Challenges of neurological assessment are presented in Table 3 and an operational workflow on when a patient should be referred to a neurologist has been added (Table S2).

## 4. Assessing clinical changes during follow-up

During follow-up, clinical features associated with disease progression versus disease control versus treatment-associated toxicity are primarily neurological. While it is recommended to capture the Karnofsky performance status (KPS) or ECOG at every visit as a rough surrogate for overall function, these measures provide little insight into the specific neurological status of patients with brain metastases.

The Response Assessment in Neuro-Oncology (RANO group) addressed some of these challenges by establishing and preliminarily validating a score card referred to as the NANO score for patients with parenchymal brain tumors [18]. The NANO assessment is supposed to be easy to perform and reproducible by non-neurologists and focuses on key items of the neurological functional status. Standardized examinations like NANO are descriptive, determine changes relative to a previous baseline, and do not include assumptions on the origin of neurological improvement or deterioration. If clinical deterioration evolves rapidly preventing formal neuroimaging assessment, clinical progression should be called only if symptoms and signs can be attributed to known preexisting brain metastasis; otherwise, differential diagnoses need to be entertained (Table 4).

## 5. Cognition and quality of life

Neuropsychological assessments are important to determine disease control as well as safety and tolerability of any treatment. A standardized test battery including Hopkins Verbal Learning Test–Revised assessing memory, Trail Making Test (TMT part A and part B) assessing visual-motor speed and executive functions, Controlled Oral Word Association Test (COWA) assessing verbal fluency and MMSE has been found useful in primary brain tumor trials and in brain metastases trials [19,20,21]. Cognitive assessments that require more than 30 min are probably not realistic, both in terms of effort for the clinical staff as well as from the patient’s perspective.

Quality of life is commonly assessed using the QLQC30 and BN20 tools in clinical trials, but these tools can also be used in clinical practice (Note S2). EQ-5D-5 L and the MD Anderson Symptom Inventory-Brain Tumor (MDASI-BT) may also be used (Table S1).

The capacity to consent should be regularly assessed during the care for patients with brain metastases [22]. Follow-up is highly recommended in a setting where there is competence for the assessment of long-term toxicity from various interventions, notably radiotherapy and cancer pharmacotherapy, ideally in a multidisciplinary setting.

## 6. Neuroimaging

Neuroimaging holds a central role in the diagnosis and follow up of patients with brain metastases. In clinical practice as well as in the context of clinical trials, solid parenchymal brain metastases are commonly distinguished from leptomeningeal metastases. An operational definition has been provided by the EANO-ESMO guidelines where solid brain metastases require a distance of 1 mm from the subarachnoid space [23].

The current RANO criteria for brain metastases have certain limitations e.g. the definition of measurable disease and the uncertainties around smaller lesions that are often readily considered for stereotactic radiosurgery. Of note, RANO proposes to assess the clinical status, steroids and imaging to determine response, however, only KPS is to be assessed, without a requirement for the performance of standard neurological examination [24]. Furthermore, clinical neurological evaluation and imaging data do not always correlate, with symptomatic small lesions close to the motor tracts or, conversely, larger lesions in functionally more silent areas.

During follow-up after radiotherapy, no validated imaging criteria have been defined. Imaging modalities other than standard MRI, such as advanced MRI sequences and FET-PET, may be useful [25]. Pseudoresponse under anti-angiogenic treatment and pseudoprogression under immunotherapy may be challenging according to RANO 2.0 [26]. The request for confirmatory scans is also problematic. It may be justified scientifically to confirm a response, e.g., for anti-angiogenic treatments, but more importantly, to request a confirmatory scan of progression in the presence of clinical deterioration is problematic, because commonly with clinical deterioration, a change of therapy will be instituted prior to confirmatory neuroimaging, precluding the detection of pseudoprogression.

## 7. Conclusions

The value of the clinical neurological assessment in patients with brain metastases in daily practice and also in the context of clinical trials needs to be recognized. Standardized and validated tools (Table S1) should be used for clinical evaluation. Discussions of complex cases should be done at a tumor board, ideally a brain metastasis-dedicated tumor board, with experts from all relevant disciplines. At treatment initiation, any neurological symptom or sign should be reported, with an estimation of their referability to individual brain metastases observed on neuroimaging. Symptomatic patients versus asymptomatic or oligosymptomatic patients should be distinguished in clinical trials as the prognosis is different. Co-medications, notably steroids, may affect the efficacy and safety of tumor-specific treatment. Clinical evaluations and CNS and extra-CNS imaging assessment should be organized in parallel during follow-up, to facilitate response assessment and clinical decision making. Clinical deterioration making imaging impossible, but clearly related to progression of CNS metastasis, should be considered sufficient to call progression. Conversely, given the broad range of neurologic impairment induced by CNS metastases directly and in response to treatment, we advocate for a holistic accounting of complications such as hemorrhage, radiation-induced necrosis, and sequelae of steroid use, and efforts at broad-scale prospective identification of patients at risk of experiencing CNS-related death.

## Supplementary Material

supplementary material

Appendix A. Supporting information

Supplementary data associated with this article can be found in the online version at doi:10.1016/j.ejca.2024.114202.

## Figures and Tables

**Table 1:** Brain metastasis-related inclusion criteria in study cohorts.

	Definition of brain metastases	Neurological symptoms	Steroids, anti-epileptic treatment	Previous local treatment to the brain
NCT00623766 [27]	At least one measurable index brain metastasis of 0.5–3 cm in diameter, or two-measurable lesions larger than 0.3 cm visible on contrast MRI, or both	Cohort A: neurologically asymptomatic	Cohort A: no systemic corticosteroid treatment in the 10 days before start of ipilimumab treatment	Previous focused radiotherapy and whole brain radiotherapy allowed if at least 14 days before start of ipilimumab. The stereotactic radiotherapy field should not have included the brain index lesion; alternatively, the lesion had to be progressive and measurable in two dimensions after any radiotherapy.
		Cohort B: symptomatically controlled brain metastases	Cohort B: concurrent systemic steroids for control of brain metastasis-related symptoms (judged by the treating physician) or edema	
BREAK-MB[28]	At least one measurable brain metastasis between 5 mm and 40 mm in diameter	Asymptomatic brain metastases	Cohort A: stable or decreasing doses of corticosteroids ongoing Cohort B: stable or decreasing doses of corticosteroids ongoing, prophylactic antiepileptic treatment allowed	Cohort A: no previous local treatment for brain metastases[28] Cohort B: disease progression in the brain after surgery, whole brain radiotherapy, or stereotactic radiosurgery
COMBI-MB[29]	Cohort A: patients with BRAFV600E mutant melanoma; target lesions could be between 0.5–4.0 cm in diameter	Cohort A: asymptomatic brain metastases	Concomitant corticosteroids must have been on a stable or decreasing dose for at least 1 month before study treatment initiation	Cohort A: patients without previous local brain-directed therapy
	Cohort B: patients with BRAFV600E mutant melanoma; target lesions could be 0.5–4.0 cm in diameter	Cohort B: asymptomatic brain metastases	Anti-epileptic therapy indicated in order to prevent neurologic symptoms caused by a pre-existing condition and not related to brain metastasis is allowed	Cohort B: patients with previous local therapy. For subjects receiving local therapy to all brain lesions (including whole brain radiotherapy), progression of preexisting lesions based on RECIST 1.1 (>20 % increase in longest diameter on baseline scan) or new measurable lesions required. For subjects receiving local (brain) therapy for some but not all lesions, disease progression based on RECIST 1.1 not required as long as there are remaining brain lesions that are measurable and not previously treated.
	Cohort C: patients with BRAFV600D/K/Rmutant melanoma, target lesions could be 0.5–4.0 cm in diameter	Cohort C: asymptomatic brain metastases		Cohort C: with or without previous local therapy
	Cohort D: patients with BRAFV600D/E/K/R-mutant melanoma; target lesions could be 0.5–4.0 cm diameter	Cohort D: symptomatic brain metastases	Concomitant corticosteroids permitted, stability of dosing not required Prophylactic or preventive anti-epileptic therapy allowed	Cohort D: with or without previous local therapy. For subjects receiving local therapy to all brain lesions (including whole-brain radiotherapy), progression of pre-existing lesions based on RECIST 1.1 (> 20 % increase in longest diameter on baseline scan) or new measurable lesions required. For subjects receiving local (brain) therapy for some but not all lesions, disease progression based on RECIST 1.1 not required as long as there are remaining brain lesions that are measurable and not previously treated.
ABC[12]	At least one target intracranial lesion of 5–40 mm	Cohort A: asymptomatic brain metastases	Not part of the inclusion criteria	Cohort A: no previous local brain therapy (surgery, stereotactic radiosurgery, or whole brain radiotherapy)
		Cohort B: asymptomatic brain metastases	Not part of the inclusion criteria	Cohort B: no previous local brain therapy (surgery, stereotactic radiosurgery, or whole-brain radiotherapy).
		Cohort C: patients who either failed local therapy, symptoms related to brain metastases, or patients with leptomeningeal disease, or any combination of these.	Not part of the inclusion criteria	Cohort C: subjects failed local therapy (ie, new brain metastases or RECIST progression in treated brain metastases with new lesions or a ≥20 % increase in sum of diameters of previously treated lesions and an absolute increase of ≥5 mm for existing lesions)
CheckMate-204 [7],[14]	At least one measurable brain metastasis (tumor diameter, 0.5 to 3 cm, as assessed by MRI that had not been previously irradiated, was not judged to require an immediate local intervention (surgery or radiosurgery)	Cohort A: no neurologic signs or symptoms	Cohort A: subjects not treated with systemic glucocorticoid therapy within 10 days before the initiation of study treatment (asymptomatic patients)	Previous stereotactic radiosurgery and excision of up to three brain metastases were permitted at least 3 weeks before study treatment, provided that neurologic sequelae had completely resolved and that measurable untreated lesions remained
		Cohort B: neurologic signs and symptoms related to metastatic intracranial lesions; no experience of seizure within 10 days prior to first treatment	Cohort B: subjects with neurologic signs and symptoms may be treated with a total daily dose of no more than 4 mg of dexamethasone that is stable or tapering for 10 days prior to first treatment; subjects with neurologic signs and symptoms who are not being treated with steroids	
NIBIT-M2[30]	BRAF wild-type or mutant melanoma with brain metastases (diameter, 5–20 mm)		Active, asymptomatic brain metastases (symptomatic brain metastases requiring immediate local intervention [radiotherapy and/or surgery], leptomeningeal disease excluded)	Not part of the inclusion criteria
Untreated brain metastases				
TRICOTEL[13]	MRI-confirmed brain metastases, 5 mm or larger in at least one dimension	Post-hoc analyses: symptomatic CNS metastases defined by medical data review according to the presence of any of the following three criteria at baseline: concomitant corticosteroids (>2 mg/day dexamethasone or equivalent); anticonvulsants; brain-related symptoms, including nausea or vomiting, headaches, seizures, motor skills dysfunction, loss of sensation, or visual or hearing deficits	No increasing corticosteroid dose during the 7 days before initiation of study treatment, or current dexamethasone or equivalent dose of more than 8 mg per day	No prior whole brain radiotherapy (previous stereotactic radiosurgery or surgical therapy of ten or fewer brain metastases was allowed)

**Legend:** CNS: central nervous system, mg: milligram, mm: millimeters, RECIST: Response Evaluation Criteria in Solid Tumours

**Table 2:** Outcome by assignment to symptomatic versus symptomatic study cohorts.

		Asymptomatic patients					Symptomatic patients				
Study (Ref)	Regimen	Number of patients	Intracranial response rate (CR+PR), n (%)	Intracranial duration of response (months)	Overall response rate, n (%)	Progression-free survival (months)	Number of patients	Intracranial response rate CR+PR), n (%)	Intracranial duration of response (months)	Overall response rate, n (%)	Progression-free survival (months)
NCT00623766 [27]	Ipilimumab	Cohort A: 51	Cohort A:8 (16 %)	Cohort A: 1.5	Cohort A: 5 (10 %)	Cohort A: 1.9	Cohort B: 21	Cohort B: 1 (5 %)	Cohort B: 1.2	Cohort B: 1 (5 %)	Cohort B: 1.2
BREAK-MB [28]	Dabrafenib	Cohort A: 172Val600Glu: 74 Val600Lys: 15	Val600Glu: 29 (39.2 %) Val600Lys: 1 (6.7 %)	Val600Glu: 20.1Val600Lys: 12.4	Val600Glu: 28 (37.8 %) Val600Lys: 0 (0 %)	Val600Glu: 16.1Val600Lys: 8.1	N/A	N/A	N/A	N/A	N/A
		Cohort B: 83Val600Glu: 65Val600Lys: 18	Val600Glu: 20 (30.8 %) Val600Lys: 4 (22.2 %)	Val600Glu: 28.1Val600Lys: 16.6	Val600Glu: 20 (30.8 %) Val600Lys: 5 (27.8 %)	Val600Glu: 16.6Val600Lys: 15.9	N/A	N/A	N/A	N/A	N/A
COMBI-MB [29]	Dabrafenib + Trametinib	Cohort A: 76	Cohort A: 44 (58 %)	Cohort A: 6.5	Cohort A: 44 (58 %)	Cohort A: 6.5	Cohort D: 17	Cohort D: 10 (59 %)	Cohort D: 4.5	Cohort D: 14 (82 %)	Cohort D: 4.5
		Cohort B: 15	Cohort B: 9 (56 %)	Cohort B: 7.3	Cohort B: 9 (56 %)	Cohort B: 12.5					
		Cohort C: 16	Cohort C: 77 (44 %)	Cohort C: 8.3	Cohort C: 7 (44 %)	Cohort C: 6.6					
ABC[12]	Ipilimumab + nivolumab Nivolumab	Cohort A: 35	Cohort A: 16 (46 %)	Cohort A: NR (2⋅9–NR)	Cohort A: N/A	Cohort A: N/A	N/A	N/A	N/A	N/A	N/A
		Cohort B: 25	Cohort B: 5 (20 %)	Cohort B: 2.5	Cohort B: N/A	Cohort B: N/A	Cohort C:16	Cohort C: 1 (6 %)	Cohort C: 2.3	Cohort C: N/A	Cohort C: N/A
CheckMate-204[7]	Ipilimumab + nivolumab	101	54 (44 %)	NR (NR-NR)	57 (57 %)	NR (32.8-NR)	18	3 (17 %)	NR (NR-NR)	4 (23 %)	NR (NR-NR)
NIBIT-M2[30]	Ipilimumab + nivolumab	27	12 (44.4 %)	8.7	12 (44.4 %)	8.7	N/A	N/A	N/A	N/A	N/A
TRICOTEL[13]	Atezolizumab + vemurafenib + cobimetinib	39	ND (38 %)	5.6	N/A	N/A	26	ND (35 %)	5.1	35 %	5.1

**Legend:** CR: complete response, N/A: not available, NR: not reached, PR: partial response

**Table 3:** Neurological assessment in the context of clinical trials.

Standardized scorecard at each assessment	
Pre-existing symptoms/signs	If yes, should be indicated at baseline as nonrelated to brain metastases, e.g., prior neuropathy
Transient deficits	Neurological assessment to be performed, but to be repeated at regular intervals until resolution of the deficit
Co-medications including steroids, pain killers, anti-epileptic drugs	The dose at each assessment should be captured. No clinical improvement should be scored if an increased dose of medication may have relieved the symptoms or signs.

**Table 4:** Interpretation of neurological deficits in patients with brain metastases.

Presentation	Likely cause	Differential diagnosis	Measures
Stable or exacerbation of preexisting symptoms or signs attributable to brain metastases	Local	Pseudoprogression	Advanced MRI, PET, surgical sampling
Stable new signs not attributable to known brain metastases	New brain metastases	Leptomeningeal disease, cerebrovascular complications, neurotoxicity from treatment	MRI, potentially including spinal MRI
New peripheral neurological symptoms and signs	Toxicity from immune checkpoint inhibition	Leptomeningeal disease, other causes of peripheral neurotoxicity	Electrophysiological work-up, cerebrospinal fluid analysis
Transient neurological deterioration	Multiple	Seizures, treatment-related toxicity, encephalopathy, cerebrovascular complications	Clinical evaluation, ancillary studies as requested by neurology
