# Supplementary material for: “Symptomatic” melanoma brain metastases: A call for clear definitions and adoption of standardized tools
Source: Eur J Cancer. Author manuscript; Available in PMC 2025 Sep 1. (PMC12290799; doi:10.1016/j.ejca.2024.114202)
Supplement: supplementary material [file NIHMS2092956-supplement-supplementary_material.docx]

**“Symptomatic” melanoma brain metastases:**

**a call for clear definitions and adoption of standardized tools**

**Supplementary material**

E. Le Rhun^1^, M. Weller^2^, C. Anders^3^, J. Larkin^4^, J. Li^5^, N. S. Moss^6^, H. Tawbi*^7^, R. Dummer*^8^

* shared co-last authors

^1^ Department of Neurosurgery, Clinical Neuroscience Center, University Hospital and University of Zurich, Zurich

^2^ Department of Neurology, Clinical Neuroscience Center, University Hospital and University of Zurich, Zurich, Switzerland

^3^ Duke Cancer Institute, Durham

^4^ The Royal Marsden NHS Foundation Trust, London, United Kingdom

^5^ Department of Radiation Oncology, University of Texas MD Anderson Cancer Center, Houston, TX, USA

**^6^** Department of Neurosurgery and Brain Metastasis Center, Memorial Sloan Kettering Cancer Center, New York, NY

^7^ Department of Melanoma Medical Oncology, Division of Cancer Medicine, The University of Texas MD Anderson Cancer Center, Houston, Texas, USA

^8^ Department of Dermatology, University Hospital and University of Zurich, Zurich, Switzerland

**Corresponding author**

Reinhard Dummer, MD, Department of Dermatology, Skin Cancer Center, University Hospital Zurich, 8091 Zurich, Switzerland

**Table S1: Tools to be considered for a standardized neurological assessment in patients with brain metastasis**

|  |  |
| --- | --- |
| Clinical assessment scorecard | NANO scale [1]  Steroid consumption |
|  |  |
| Cognitive assessment tests | Hopkins Verbal Learning Test–Revised [2]  Trail Making Test (TMT part A and part B) [3]  Controlled Oral Word Association Test (COWA) [4]  MMSE (at baseline) [5] |
|  |  |
| Health-related quality of life questionnaires | EORTC QLQ-C30 [6]  EORTC QLQ-BN20 [7] |
|  |  |
| Imaging assessment | No validated scorecard available.  Standardized response criteria, such as RANO-BM [8] should be used. Central review is recommended. |

**Table S2: When should my patient see a neurologist?**

| When should my patient see a neurologist? |
| --- |
| My patient is experiencing new neurological deficits, and I do not know why.  The neurological symptoms and signs are fluctuating and I am not sure this is related to an epileptic seizure.  I am not sure whether the neurological problems of my patient are related to prior therapy.  I have difficulty to determine whether the neurological symptoms and signs correspond to the lesions that are visible on the brain MRI.  I do not know how to handle the neurological complains from my patient.  My patient has a medical history of neurological disease which needs a follow-up. |

**Note S1**

The BREAK-MB explored the activity of dabrafenib in asymptomatic patients only with a stable or decreasing dose of steroids [9], and the NIBIT-M2 trial explored the activity of nivolumab combined with ipilimumab in patients with active, asymptomatic brain metastases but no specification on steroid use [10] (**Tables 1 and 2**). Cohorts of patients with symptomatic brain metastases of melanoma were planned to be included in most trials [11] [12] [13] [14] [15].

The first clinical trial to assess immune checkpoint inhibition in patients with melanoma brain metastases examined single agent ipilimumab at 10 mg/kg and included 2 cohorts. The first cohort was defined by the absence of neurological symptoms or need for corticosteroids, the second defined “symptomatic” patients by the need to maintain a stable “dose of brain-metastases-related” corticosteroids at the time of initiation of therapy [13]. This seminal trial indicated that while ipilimumab had intracranial activity comparable to extracranial activity in asymptomatic patients, it had virtually no activity in patients receiving steroids.

COMBI-MB investigated the combination of dabrafenib and trametinib in three cohorts of patients with asymptomatic B-RAF-mutant brain metastases and included a fourth cohort of 17 patients that were “symptomatic” [14]. In the primary report, this combination of treatment seemed to have a similar clinical activity among the different studied groups (44-59% of response rate), but with shorter duration of response in symptomatic melanoma brain metastases.

The ABC trial examined the role of nivolumab alone or of the combination of nivolumab and ipilimumab in patients with asymptomatic melanoma brain metastases. A third cohort, cohort C, assessed single agent anti-PD1 in “symptomatic” patients that had either neurologic symptoms, required steroids, or had leptomeningeal disease [11]. Nivolumab had very limited activity in this population with an intracranial response rate of ~6% (one of 16 patients).

CheckMate-204 investigated the combination of ipilimumab and nivolumab in patients with melanoma brain metastases who were either asymptomatic (cohort A) or symptomatic (cohort B). It reported a high rate (57%) of durable responses in asymptomatic patients. However, the combination had only a 17% intracranial response rate in the small cohort of patients that were defined as “symptomatic” by having neurological symptoms or requiring up to 4 mg of dexamethasone [16] [15].

A post-hoc analysis of asymptomatic *versus* symptomatic patients with brain metastases was performed in the TRICOTEL trial assessing vemurafenib plus cobimetinib combined with atezolizumab [12] . Of the 11 patients treated with a dexamethasone equivalent dose of 2 mg or more at baseline, the dose was tapered or discontinued in 4 patients during the first cycle of vemurafenib plus cobimetinib, before atezolizumab initiation.

**Note S2**

The EORTC QLQ-C30 includes five functional items (physical, role, emotional, social and cognition), three symptom items (fatigue, nausea and vomiting and pain), a global health status scale, and six single items (dyspnea, insomnia, appetite loss, constipation, diarrhea and financial difficulties) [34]. The EORTC QLQ-BN20 questionnaire comprises four symptom items (future uncertainty, visual disorder, motor dysfunction, and communication deficit) and seven single items headaches, seizures, drowsiness, hair loss, itchy skin, weakness of legs and bladder control [35].

**References**

[1] Nayak L, DeAngelis LM, Brandes AA, Peereboom DM, Galanis E, Lin NU, et al. The Neurologic Assessment in Neuro-Oncology (NANO) scale: a tool to assess neurologic function for integration into the Response Assessment in Neuro-Oncology (RANO) criteria. Neuro-Oncology 2017;19:625–35. https://doi.org/10.1093/neuonc/nox029.

[2] Benedic Ralph H B, Schretlen, David, Groninger, Lowell, Brandt, Jason. Hopkins Verbal Learning Test – Revised: Normative Data and Analysis of Inter-Form and Test-Retest Reliability. The Clinical Neuropsychologist 1998:Vol. 12, No. 1, pp. 43–55.

[3] Reitan RM. Validity of the Trail Making Test as an Indicator of Organic Brain Damage. Percept Mot Skills 1958;8:271–6. https://doi.org/10.2466/pms.1958.8.3.271.

[4] Benton, A. I. Multilingual Aphasia Examination 1989.

[5] Folstein MF, Folstein SE, McHugh PR. “Mini-mental state”. A practical method for grading the cognitive state of patients for the clinician. Journal of Psychiatric Research 1975;12:189–98. https://doi.org/10.1016/0022-3956(75)90026-6.

[6] Aaronson NK, Ahmedzai S, Bergman B, Bullinger M, Cull A, Duez NJ, et al. The European Organization for Research and Treatment of Cancer QLQ-C30: a quality-of-life instrument for use in international clinical trials in oncology. J Natl Cancer Inst 1993;85:365–76. https://doi.org/10.1093/jnci/85.5.365.

[7] Taphoorn MJB, Claassens L, Aaronson NK, Coens C, Mauer M, Osoba D, et al. An international validation study of the EORTC brain cancer module (EORTC QLQ-BN20) for assessing health-related quality of life and symptoms in brain cancer patients. Eur J Cancer 2010;46:1033–40. https://doi.org/10.1016/j.ejca.2010.01.012.

[8] Lin NU, Lee EQ, Aoyama H, Barani IJ, Barboriak DP, Baumert BG, et al. Response assessment criteria for brain metastases: proposal from the RANO group. Lancet Oncol 2015;16:e270-278. https://doi.org/10.1016/S1470-2045(15)70057-4.

[9] Long GV, Trefzer U, Davies MA, Kefford RF, Ascierto PA, Chapman PB, et al. Dabrafenib in patients with Val600Glu or Val600Lys BRAF-mutant melanoma metastatic to the brain (BREAK-MB): a multicentre, open-label, phase 2 trial. Lancet Oncol 2012;13:1087–95. https://doi.org/10.1016/S1470-2045(12)70431-X.

[10] Di Giacomo AM, Chiarion-Sileni V, Del Vecchio M, Ferrucci PF, Guida M, Quaglino P, et al. Primary Analysis and 4-Year Follow-Up of the Phase III NIBIT-M2 Trial in Melanoma Patients With Brain Metastases. Clin Cancer Res 2021;27:4737–45. https://doi.org/10.1158/1078-0432.CCR-21-1046.

[11] Long GV, Atkinson V, Lo S, Sandhu S, Guminski AD, Brown MP, et al. Combination nivolumab and ipilimumab or nivolumab alone in melanoma brain metastases: a multicentre randomised phase 2 study. Lancet Oncol 2018;19:672–81. https://doi.org/10.1016/S1470-2045(18)30139-6.

[12] Dummer R, Queirolo P, Gerard Duhard P, Hu Y, Wang D, de Azevedo SJ, et al. Atezolizumab, vemurafenib, and cobimetinib in patients with melanoma with CNS metastases (TRICOTEL): a multicentre, open-label, single-arm, phase 2 study. Lancet Oncol 2023:S1470-2045(23)00334-0. https://doi.org/10.1016/S1470-2045(23)00334-0.

[13] Margolin K, Ernstoff MS, Hamid O, Lawrence D, McDermott D, Puzanov I, et al. Ipilimumab in patients with melanoma and brain metastases: an open-label, phase 2 trial. Lancet Oncol 2012;13:459–65. https://doi.org/10.1016/S1470-2045(12)70090-6.

[14] Davies MA, Saiag P, Robert C, Grob J-J, Flaherty KT, Arance A, et al. Dabrafenib plus trametinib in patients with BRAFV600-mutant melanoma brain metastases (COMBI-MB): a multicentre, multicohort, open-label, phase 2 trial. Lancet Oncol 2017;18:863–73. https://doi.org/10.1016/S1470-2045(17)30429-1.

[15] Tawbi HA, Forsyth PA, Algazi A, Hamid O, Hodi FS, Moschos SJ, et al. Combined Nivolumab and Ipilimumab in Melanoma Metastatic to the Brain. N Engl J Med 2018;379:722–30. https://doi.org/10.1056/NEJMoa1805453.

[16] Tawbi HA, Forsyth PA, Hodi FS, Algazi AP, Hamid O, Lao CD, et al. Long-term outcomes of patients with active melanoma brain metastases treated with combination nivolumab plus ipilimumab (CheckMate 204): final results of an open-label, multicentre, phase 2 study. Lancet Oncol 2021;22:1692–704. https://doi.org/10.1016/S1470-2045(21)00545-3.
